# Increased Autoimmunity Burden Is a Risk Factor for Developing Irritable Bowel Syndrome-Like Symptoms in Quiescent Inflammatory Bowel Disease

**DOI:** 10.1007/s10620-025-09633-4

**Published:** 2026-01-03

**Authors:** Andrew Chang, Bita Shahrvini, Janice Oh, Divya P. Prajapati, Mark Baniqued, Rhett Harmon, Alexandra C. Greb, Nirupama Bonthala, Jenny S. Sauk, Andrea Shin, Lin Chang, Berkeley N. Limketkai

**Affiliations:** 1 Department of Medicine, UCLA School of Medicine, Los Angeles, CA USA; 2 Vatche and Tamar Manoukian Division of Digestive Diseases, UCLA School of Medicine, Los Angeles, CA USA; 3 Department of Medicine, Olive View UCLA, Sylmar, CA USA

**Keywords:** Inflammatory bowel disease, Irritable bowel syndrome, Irritable bowel syndrome-like symptoms, Autoimmune comorbidities

## Abstract

**Background:**

A significant proportion of patients with inflammatory bowel disease (IBD) continue to experience gastrointestinal symptoms despite achieving endoscopic remission. These irritable bowel syndrome (IBS)-like symptoms may result from gut-brain axis dysfunction or low-grade immune activation.

**Aims:**

We aimed to determine whether extra-intestinal autoimmune comorbidities are associated with IBS-like symptoms in quiescent IBD.

**Methods:**

We conducted a retrospective cohort study of adult patients with IBD with endoscopic remission, excluding patients with prior IBS or less than 12 months of follow-up. The primary outcome was the development of IBS-like symptoms within 12 months of baseline colonoscopy. Multivariable Cox regression and Kaplan–Meier survival analysis were used to identify risk factors and assess time to symptom development.

**Results:**

Among 399 patients, 80 (20.1%) developed new IBS-like symptoms within one year of achieving endoscopic remission. Mean time to symptom onset was 169.2 days (SD 110.3). Symptom development was significantly associated with extra-intestinal autoimmune comorbidities (HR 2.05; 95% CI 1.15–3.66; *p* = 0.015) and anxiety (HR 1.87; 95% CI 1.13–3.12; *p* = 0.016). Kaplan–Meier analysis showed that patients with autoimmune comorbidities had a significantly higher cumulative incidence of IBS-like symptoms compared to those without (log-rank *p* < 0.001).

**Conclusion:**

Among patients with IBD with endoscopic remission at baseline, 20.1% developed IBS-like symptoms within one year. The presence of autoimmune comorbidity was significantly associated with earlier development of IBS-like symptoms. These findings suggest that immune system dysregulation may play a role in persistent gastrointestinal symptoms and highlight the need for further research into the pathophysiology of IBS-like symptoms in quiescent IBD.

**Supplementary Information:**

The online version contains supplementary material available at 10.1007/s10620-025-09633-4.

## Introduction

Inflammatory bowel disease (IBD) is a chronic immune-mediated condition of the gastrointestinal tract that commonly presents with abdominal pain and altered bowel habits. Historically, IBD specialists aimed for clinical or symptomatic remission as the treatment target [1, 2]. As newer and more effective therapeutic options became available to treat IBD, the treatment target has evolved to aiming for endoscopic remission, to reduce the chance of relapse [3].

Despite achieving endoscopic remission, 23.5% of patients will still experience residual irritable bowel syndrome (IBS)-like symptoms, such as abdominal pain and altered bowel habits [4]. Although they may not formally meet the Rome IV criteria for IBS, gastrointestinal symptoms (e.g., abdominal discomfort, altered bowel habits, and change in stool consistency) are shared by both conditions. This overlap has led researchers to investigate whether the underlying mechanisms of these symptoms are similar to those of IBS. One potential contributor is a perturbation in the gut–brain axis. Studies have shown that IBS-like symptoms are strongly associated with patients with psychiatric comorbidities [4, 5]. Given the high prevalence of anxiety and depression among patients with IBD and the role of the gut–brain axis in IBS, this association is not surprising and is a likely contributor to IBS-like symptoms [6].

Another plausible contributor to the presence of residual gastrointestinal symptoms despite evidence of biochemical, endoscopic, and histologic control of inflammation is the persistence of low-grade inflammation in the gut or nervous system that is otherwise not detectable. This hypothesis is supported by studies showing that patients with IBS without evidence of inflammation with conventional tests may still have increased immune system activity in the gut [7]. Additionally, histologic studies have shown that patients with IBS exhibit similar levels of immune cell infiltration in the gut compared to patients with quiescent IBD, both of which are elevated compared to healthy controls [8]. Moreover, it has been shown that autoimmune diseases are associated with the diagnosis of IBS [9]. Given these observations, the aim of this study was to determine whether patients with quiescent IBD and diagnosed with extra-intestinal autoimmune diseases—presumably possessing a higher overall inflammatory burden—were at higher risk for developing IBS-like symptoms after achieving endoscopic remission compared to those without extra-intestinal autoimmune diseases.

## Materials and Methods

### Study Design and Patient Population

This retrospective longitudinal cohort study included non-consecutive adult patients with IBD who received care at the ambulatory gastrointestinal clinics of a large tertiary-care academic center and who were in symptomatic and endoscopic remission at the time of index colonoscopy, performed between January 2014 and September 2023. Index colonoscopy was defined as the first colonoscopy with no evidence of inflammatory changes visualized throughout the entire colon and terminal ileum. Patients with a diagnosis of IBS prior to this index colonoscopy or who had less than 12 months of post-colonoscopy follow-up were excluded from this study.

### Identification of IBS-Like Symptoms

IBS-like symptoms were identified through chart review of gastroenterology provider notes during the first year following index colonoscopy. IBS-like symptoms were defined according to the Rome IV criteria for IBS, except that symptoms were required to be persistent, without the Rome IV requirement of occurring at least one day per week for the past three months. Patients with any evidence of active IBD, such as elevated fecal calprotectin, elevated C-reactive protein, or inflammatory changes on a repeat colonoscopy, were excluded from the IBS-like symptom group.

### Variable Abstraction

Variables abstracted through chart review for univariate analysis included medical comorbidities, autoimmune comorbidities, history of anxiety disorder, history of depression, prior surgeries, history of substance use disorder, prior IBD medications, and histologic remission at the time of index colonoscopy. Autoimmune comorbidities were identified through review of the patient’s problem list in the electronic health record and included, but were not limited to type 1 diabetes, systemic lupus erythematosus, rheumatoid arthritis, multiple sclerosis, Graves’ disease, Hashimoto’s thyroiditis, Sjögren’s syndrome, ankylosing spondylitis, IgA nephropathy, vitiligo, scleroderma, psoriasis, celiac disease, and autoimmune hepatitis. Chi-squared tests or Student’s t-tests were performed to assess the association between these variables and the development of new IBS-like symptoms within one year after index colonoscopy.

### Statistical Analysis

We then performed multivariable Cox regression to further examine the risk of developing new IBS-like symptoms, while adjusting for age, sex, body mass index (BMI), IBD type, depression, anxiety, history of biologic therapy, history of immunomodulators, and histologic remission status at the time of index colonoscopy. We also performed multivariable Cox regression to calculate the hazard ratio for developing IBS-like symptoms among individual autoimmune comorbidities, while also adjusting for the above variables. Kaplan–Meier survival analysis was used to visualize time to IBS-like symptoms within the one-year follow-up period, stratified by the presence or absence of extra-intestinal autoimmune comorbidity. Statistical analysis was conducted using Stata 18.0 (College Station, Texas).

## Results

### Patient Characteristics

The study included 399 patients who met the inclusion criteria (Table 1). The mean age of patients was 42.8 years (standard deviation [SD] 14.8), and 205 (51.4%) were female. Most patients were white (75.9%) and non-Hispanic (67.9%). Ulcerative colitis (UC) accounted for 215 (53.9%) of patients, Crohn’s disease (CD) accounted for 178 (44.6%), and indeterminate colitis accounted for the remaining 6 (1.5%). Ninety-six (24.1%) patients were diagnosed with depression, and 132 (33.1%) patients were diagnosed with anxiety. A majority of patients had a history of using immunomodulators (56.1%) and biologic therapy (63.7%).

**Table 1:** Baseline demographic and clinical characteristics of patients with quiescent IBD, stratified by development of IBS-like symptoms

	Developed IBS-Like Symptoms (*n* = 80)	Did Not Develop IBS-Like Symptoms (*n* = 319)	Overall	*p* value
Age, mean years (SD)	41.5 (15.4)	43.2 (14.6)	42.8 (14.8)	0.359
Sex				
Female	48 (60%)	157 (49.2%)	205 (51.4%)	0.084
Male	32 (40%)	162 (50.8%)	194 (48.6%)	
Race				
Caucasian	69 (86.3%)	234 (73.4%)	303 (75.9%)	0.117
African American	0 (0%)	14 (4.4%)	14 (3.5%)	
Asian	4 (5%)	31 (9.7%)	35 (8.8%)	
Unknown	7 (8.8%)	40 (12.5%)	47 (11.8%)	
Ethnicity				
Non-Hispanic	67 (83.8%)	265 (83.1%)	271 (67.9%)	0.984
Hispanic	7 (8.8%)	30 (9.4%)	98 (24.6%)	
Unknown	6 (7.5%)	24 (7.5%)	30 (7.5%)	
BMI (SD)	27.0 (6.1)	25.6 (5.3)	25.9 (5.5)	0.055
IBD classification				
Crohn’s disease	41 (51.3%)	137 (42.9%)	178 (44.6%)	0.255
Ulcerative colitis	37 (46.3%)	178 (55.8%)	215 (53.9%)	
Indeterminate colitis	2 (2.5%)	4 (1.3%)	6 (1.5%)	
Depression comorbidity present	31 (38.8%)	65 (20.4%)	96 (24.1%)	0.001
Anxiety comorbidity present	41 (51.3%)	94 (29.5%)	132 (33.1%)	< 0.001
Autoimmune comorbidity present	17 (21.3%)	26 (8.2%)	43 (10.8%)	0.001
History of biologic therapy usage	52 (65%)	202 (63.3%)	254 (63.7%)	0.780
History of immunomodulator usage	46 (57.5%)	178 (55.8%)	224 (56.1%)	0.784
Histologic remission at time of negative colonoscopy	60 (75%)	236 (74.0%)	296 (74.2%)	0.852
Prior intestinal surgical history	9 (11.3%)	38 (11.9%)	47 (11.8%)	0.87

### Risk Factors for IBS-Like Symptoms

Among all patients, 80 (20.1%) developed new IBS-like symptoms within one year of achieving endoscopic remission. In univariate analysis, age, sex, race, ethnicity, IBD subtype, prior use of biologic therapy, prior use of immunomodulators, and histologic remission were not associated with the development of IBS-like symptoms. Chronic depression and anxiety were significantly associated with IBS-like symptoms (*p* = 0.001 and *p* < 0.001, respectively). Autoimmune comorbidities were also significantly associated with the development of IBS-like symptoms (*p* = 0.001). In multivariable Cox regression analysis, autoimmune comorbidities were significantly associated with the development of IBS-like symptoms (HR 2.05; 95% CI 1.15–3.66; *p* = 0.015) (Table 2). Anxiety comorbidity was also statistically significant (HR 1.87; 95% CI 1.13–3.12; *p* = 0.016), but depression comorbidity was not.

**Table 2:** Multivariable cox regression identifying independent risk factors for development of IBS-like symptoms

	Hazard ratio (95% CI)	*p* value
Age	0.99 (0.97–1.00)	0.155
Sex	1.17 (0.73–1.87)	0.514
BMI	1.03 (1.00–1.07)	0.063
IBD classification	0.84 (0.54–1.31)	0.447
Depression comorbidity present	1.46 (0.86–2.48)	0.160
Anxiety comorbidity present	1.87 (1.13–3.12)	0.016
Autoimmune comorbidity present	2.05 (1.15–3.66)	0.015
History of biologic therapy usage	0.79 (0.47–1.34)	1.340
History of immunomodulator usage	1.10 (0.67–1.81)	1.810
Histologic remission at time of negative colonoscopy	1.00 (0.60–1.67)	1.670

### Subgroup Analysis of Individual Autoimmune Comorbidities

We then performed subgroup analyses of individual autoimmune comorbidities (Table 3). Conditions reported in fewer than three patients were excluded from this analysis. Of the 43 patients with autoimmune comorbidities, psoriasis was the most common (*n* = 16), followed by rheumatoid arthritis (*n* = 5). Having rheumatoid arthritis (HR 4.99; 95% CI 1.43–17.39; *p* = 0.012) was significantly associated with the development of IBS-like symptoms. No other autoimmune comorbidities showed a significant association.

**Table 3:** Hazard ratios for developing IBS-like symptoms across individual extra-intestinal autoimmune conditions

Autoimmune Condition	Total (*n* = 43)	Hazard ratio (95% CI)	*p* value
Any extra-intestinal autoimmune condition	43	2.05 (1.15–3.66)	0.015
Lupus	4	1.02 (0.14–7.44)	0.987
Rheumatoid arthritis	5	4.99 (1.43–17.39)	0.012
Psoriasis	16	1.76 (0.76–4.10)	0.187
Autoimmune hepatitis	3	3.34 (0.78–14.42)	0.105
Other	17		

### Kaplan–Meier Analysis of Symptom Onset

**Fig. 1:**
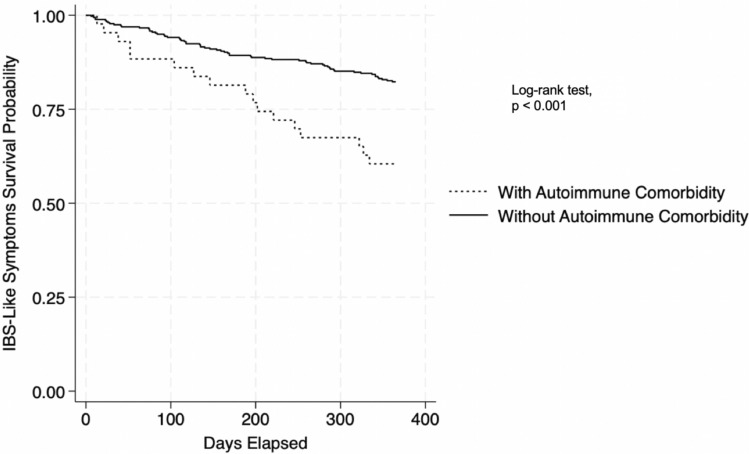
Kaplan–Meier curve showing time to development of ibs-like symptoms, stratified by presence of autoimmune comorbidity

The timing of symptom development was also analyzed using Kaplan–Meier survival curves. Among the 80 patients who developed IBS-like symptoms, the mean time to symptom onset was 169.2 days (SD 110.3). Kaplan–Meier curves showed that patients with autoimmune comorbidities had a significantly higher cumulative probability of IBS-like symptoms compared to those without (log-rank *p* < 0.001) (Fig. 1). Among those who did experience IBS-like symptoms, there was no significant difference in time to onset for those with versus without autoimmune comorbidities (*p* = 0.94).

### Biologic Therapy Use and Escalation

Finally, we compared biologic therapy use between patients who developed IBS-like symptoms and patients who did not. The proportion of patients on biologics at time of index colonoscopy was similar between groups (*p* = 0.705), as was the proportion on biologics at 12-month follow-up (*p* = 0.521) (Supplemental Table 1). In contrast, escalation of therapy, defined as either initiation of a biologic in patients not previously on therapy or a change in biologic agent, occurred more frequently in patients who developed IBS-like symptoms than in those without (*p* = 0.002).

## Discussion

This study found that 20.1% of quiescent patients with IBD developed IBS-like symptoms within 1 year of achieving endoscopic remission. Our study also identified increased autoimmune burden as a significant risk factor for developing IBS-like symptoms in patients with quiescent IBD, which to our knowledge is a novel finding. Previous studies have shown that IBS is linked with autoimmune diseases, so it is not surprising that we observed a similar association in patients with quiescent IBD [9, 10].

The finding that 20.1% of patients with quiescent IBD experience IBS-like symptoms is consistent with a previous meta-analysis reporting a prevalence of 23.5% [4]. Our study further confirms that stricter criteria of endoscopic remission result in a lower prevalence compared to the previously reported 33.6% for patients who achieved clinical remission. Unlike what was shown in previous studies, our study did not find a statistically significant association between sex and development of IBS-like symptoms, although there was a numerically greater proportion of women who developed IBS-like symptoms, and the p value approached statistical significance [11]. IBS has been strongly linked to female sex, with women reporting greater symptom severity and increased healthcare utilization related to functional disorders. One possible explanation for this lack of statistical significance is that other factors (e.g., psychiatric comorbidities, autoimmune comorbidities) have stronger influence than sex-based factors, such as hormonal differences, on the development of IBS-like symptoms. A moderate sample size may have also limited our power to detect a statistical difference. Additional studies with larger cohorts may be helpful in clarifying the role of sex in this population.

Although biologic use at baseline and at 12-month follow-up did not differ between patients who developed IBS-like symptoms and those who did not, escalation of biologic therapy was more frequent in patients with IBS-like symptoms. Medication use was assessed at the 12-month follow-up for all patients, rather than at the exact time of IBS-like symptom onset, to allow for a consistent comparison between groups. While we report biologic use and changes, the study was not powered to assess causal effects of specific medications on IBS-like symptom development. Therefore, we cannot conclude whether treatment escalation contributed to symptom development or vice versa, and prospective studies are needed to clarify this relationship.

Given the high proportion of patients who suffer IBS-like symptoms despite achieving IBD remission, as well as the increase in resource utilization resulting from IBS-like symptoms, there has been more investigation into the root causes of these residual symptoms [12]. Although the ultimate etiology is likely multifactorial, key contributors identified to date include alterations in the gut–brain axis, visceral hypersensitivity, gut dysbiosis, and chronic low-grade inflammation. Increased autoimmune burden may amplify these processes through several pathways. First, even when IBD is clinically and endoscopically in remission, poorly controlled extra-intestinal autoimmune diseases can lead to persistent immune system activation. This manifests as increased systemic circulation of proinflammatory cytokines, which can affect the gut. Second, the presence of multiple autoimmune diseases implies a “more dysregulated” immune system. The combination of these two can then result in low-grade inflammation of the gut or the nervous system. This likely occurs through a mechanism similar to patients with IBS, where the low-grade inflammation results in increased mast cells and T cells in the intestinal mucosa [13, 14]. This subsequently leads to visceral hypersensitivity through sensitizing local nerve endings and disrupting gut sensation, which is responsible for the increase in IBS-like symptoms [15]. As is the case for patients with IBS, these inflammatory changes can occur even in the absence of overt histologic abnormalities or biochemical evidence of inflammation. In our study, we found no significant association between histologic remission at baseline and the presence of IBS-like symptoms. This could be due to sampling limitations, as mucosal biopsies may miss patchy or deep-layer inflammation.

An altered gut microbiome has a strong association with IBS, so it is unsurprising that they may also contribute to these IBS-like symptoms [16]. In our population, gut dysbiosis is also multifactorial. Despite achieving remission, patients with IBD can still experience decreased diversity of gut bacteria, decreased proportion of beneficial bacteria, and an overgrowth of pathogenic bacteria that can contribute to the bloating, abdominal discomfort, and changes in bowel habits found in patients with IBS-like symptoms [17]. Nonetheless, gut dysbiosis has also been linked to multiple extra-intestinal autoimmune diseases [18, 19]. These autoimmune diseases will share the same underlying mechanisms, such as immune system dysregulation, which can disrupt the gut microbiome and contribute to the gastrointestinal symptoms.

Prior research has highlighted other contributors, including psychiatric comorbidities, which aligns with the well-established connection between mood disorders and IBS, as well as emerging insights into the gut–brain axis. Our study did confirm an association between anxiety disorder and developing IBS-like symptoms, but not for depression. The lack of association between depression and developing IBS-like symptoms could be due to the collinearity of depression with anxiety, which may have obscured its independent effect in the multivariate model. The proportion of patients who developed new IBS-like symptoms is consistent with prior studies who used endoscopic remission as their inclusion criteria [4].

One of the strengths of our study is that we only included patients who had endoscopy-proven remission, which is a more objective measure of inactive disease than symptoms and biochemical markers. However, several limitations apply to our study and should be considered while interpreting the results. First, our study was a retrospective analysis, which limits our ability to establish a causal relationship between the variables. Second, our study was a single-center study at a large tertiary-care academic health system, which could affect the generalizability of our findings to other populations. Third, the sample sizes for each individual autoimmune disease were relatively small (Supplemental Table 2). This led to large standard deviations and the difficulty to draw disease-specific conclusions. Additionally, our study was not powered to detect potential confounding effects of BMI on the development of IBS-like symptoms, and dietary data were not available for analysis. Thus, we cannot exclude the possibility that differences in metabolic factors may have contributed to the observed association. Moreover, our data were collected through chart review of available physician notes, which were not systematically designed to capture IBS-like symptoms post-remission. As a result, symptom documentation likely varied across clinical encounters and patients, introducing the potential for measurement bias. Lastly, we only assessed outcomes within a one-year timeframe, which may have limited our ability to detect delayed or evolving development of IBS-like symptoms.

In conclusion, this study demonstrated that the development of IBS-like symptoms is prevalent even in patients with quiescent IBD and no history of IBS at baseline. This study identified psychiatric comorbidity and increased autoimmunity burden as risk factors for the development of IBS-like symptoms within 1 year of achieving endoscopy-proven IBD remission. These findings suggest that patients with autoimmune diseases, as well as those with psychiatric comorbidities, may benefit from closer monitoring or be counseled on self-monitoring for the development of IBS-like symptoms, even after achieving IBD remission. This finding prompts additional studies regarding the underlying mechanisms that lead to these findings. Future research should include prospective studies to better assess this relationship and whether control of autoimmune comorbidities reduces the risk of developing IBS-like symptoms.

## Supplementary Information

Below is the link to the electronic supplementary material.

Supplementary file1 (DOCX 13 KB)

## Data Availability

All data supporting the findings of this study are included within the manuscript and its Supplementary Information. Individual-level patient data are not publicly available due to privacy regulations but may be made available from the corresponding author upon reasonable request and with appropriate institutional approvals.

## Declarations

Jenny S. Sauk has served as a Consultant for Abbvie, CorEvitas, Celltrion, and Genentech.
