# Supplementary material for: Increased Autoimmunity Burden Is a Risk Factor for Developing Irritable Bowel Syndrome-Like Symptoms in Quiescent Inflammatory Bowel Disease
Source: Dig Dis Sci. 2026 Jan 3;71(6):2340–6. doi: 10.1007/s10620-025-09633-4 (PMC13357454; doi:10.1007/s10620-025-09633-4)
Supplement: Supplementary file 1 — Supplementary file1 (DOCX 13 KB) [file 10620_2025_9633_MOESM1_ESM.docx]

**Supplemental Table 1. Proportions of Biologic Therapy Use and Escalation in Patients with Versus without IBS-Like Symptoms at 12-Month Follow-Up**

|  | **Developed IBS-Like Symptoms** | **Did Not Develop IBS-Like Symptoms** | **p value** |
| --- | --- | --- | --- |
| **Baseline Biologic** | 0.63 | 0.60 | 0.705 |
| **Biologic at 12 Months** | 0.66 | 0.62 | 0.521 |
| **Biologic Escalation** | 0.14 | 0.04 | 0.002 |

**Supplemental Table 2. Number of Patients per Autoimmune Condition**

| **Autoimmune Condition** | **Total** |
| --- | --- |
| Psoriasis | 16 |
| Rheumatoid Arthritis | 5 |
| Lupus | 4 |
| Autoimmune Hepatitis | 3 |
| Hashimoto’s Thyroiditis | 2 |
| Type 1 Diabetes | 2 |
| Multiple Sclerosis | 2 |
| Alopecia Areata | 2 |
| Primary Sclerosing Cholangitis | 2 |
| Vitiligo | 1 |
| Antiphospholipid Syndrome | 1 |
| Sjogren’s Disease | 1 |
| Alopecia Areata | 1 |
| Ankylosing Spondylitis | 1 |
| IgA Nephropathy | 1 |
| Grave’s Disease | 1 |
